# A comprehensive systematic review of health-related quality of life measures in short stature paediatric patients

**DOI:** 10.1007/s12020-024-03938-6

**Published:** 2024-07-17

**Authors:** Adekunle Adedeji, Stefanie Witt, Florian Innig, Julia Quitmann

**Affiliations:** 1 Department of Social Work, Hamburg University of Applied Sciences, Hamburg, Germany; 2 Department of Medical Psychology, University Medical Center, Hamburg-Eppendorf, Hamburg, Germany; 3 BKMF Federal Association for People of Short Stature and their Families (Bundesverband Kleinwüchsige Menschen und ihre Familien e.V.), BKMF, Hamburg, Germany

**Keywords:** PROMs, Patient report, Proxy report, Quality of life, Short stature paediatric patients

## Abstract

This systematic review investigates Patient-reported Outcome Measures (PROMs) and Observed Reported Outcome Measures (ObsROMs) pertinent to assessing Health-Related Quality of Life (HRQoL) in short-stature paediatric patients, focusing on Achondroplasia (ACH), Growth Hormone Deficiency (GHD), Isolated Growth Hormone Deficiency (IGHD), and Small-for-Gestational-Age (SGA) diagnoses. Utilising rigorous selection criteria, 53 studies published from 1998 to 2023 were analysed, revealing a predominance of European-based research. Notably, the review elucidated the utilisation of disease-specific and generic HRQoL measures, showcasing the multifaceted nature of short-stature conditions and their impact across physical, emotional, and social domains. The Quality of Life in Short Stature Youth (QoLISSY), Paediatric Quality of Life Inventory (PedsQL), and KIDSCREEN emerged as frequently employed instruments, offering nuanced insights into HRQoL perceptions across diverse age demographics. Additionally, the review highlighted the adaptation of adult HRQoL measures for adolescent populations, signalling a need for age-appropriate assessment tools. Furthermore, integrating PROMs and ObsROMs in HRQoL assessment underscored a comprehensive approach, considering both subjective patient perspectives and observed outcomes. Future research directions encompass comprehensive search strategies, longitudinal studies with diverse populations, and the development of age-appropriate HRQoL assessment tools. In conclusion, this review emphasises the importance of comprehensive HRQoL assessment to address the diverse needs of short-stature paediatric patients effectively.

## Background

Short stature in paediatric patients presents a multifaceted challenge beyond physical health concerns, encompassing profound implications for psychological, social, and emotional well-being. Understanding the intricate interplay between short stature and health-related quality of life (HRQoL) in children and adolescents is imperative for tailoring effective clinical interventions and implementing comprehensive care strategies [1, 2]. This systematic review aims to explore the extensive body of research on HRQoL measures among paediatric patients with short stature, examining a spectrum of perspectives ranging from generic to specific assessments across diverse age groups. It will further explore the use of patient-reported and observer-measured outcomes to provide perspective on the impact of short stature on HRQoL.

Stature is a hereditary trait controlled by both genetics and environmental factors. Short stature is defined as the height of an individual two standard deviations (SD) below the mean height of the population of the same age and sex. Short stature has been shown to have a negative impact on the HrQoL of children and adolescents [3–5]. In addition, short stature has been associated with a variety of adverse outcomes that extend beyond stature itself, such as lower social competencies [6], stigmatisation and bullying [3, 7], impaired psychosocial well-being [8], and depression [9].

For the current review, we focus on diagnoses such as Growth hormone deficiency (GHD), Isolated Growth Hormone Deficiency (IGHD), Small-for-gestational-age (SGA), and Achondroplasia (ACH). Growth hormone deficiency (GHD) is a rare disorder caused by insufficient growth hormone [10]. It is characterised by the inadequate secretion of growth hormone from the anterior pituitary gland. GHD can be present at birth (congenital) or develop later (acquired). Children with GHD have abnormally short stature with normal body proportions [10]. Similarly, in IGHD, the pituitary gland produces insufficient growth hormone, leading to short stature and delayed growth in children. It typically presents with normal body proportions and can be diagnosed through growth charts, hormone tests, and MRI scans. While IGHD is specifically characterised by a deficiency in growth hormone alone, GHD can refer to a broader condition where growth hormone deficiency may occur alongside other pituitary hormone deficiencies. Small-for-gestational-age (SGA) short stature describes children born with birth weight and length below the 10th percentile for their gestational age and sex [11]. This means that they are smaller than usual for the number of weeks of pregnancy. SGA can be caused by various factors, including genetic, maternal, placental, and foetal factors [11]. Achondroplasia is a genetic disorder affecting bone growth and dwarfism [12]. It is caused by a mutation in the Fibroblast Growth Factor Receptor 3 (FGFR3) gene, leading to abnormal bone growth in the cartilage of the growth plate [12]. The condition is inherited in an autosomal dominant pattern, which means that a child only needs to inherit one copy of the altered gene from one parent to develop the disorder.

Reviewing HRQoL measures in paediatric patients with short stature across different diagnoses highlights shared challenges children with short stature face regarding social, psychological, and physical difficulties, such as bullying, low self-esteem, and practical limitations. Understanding how this is measured across different diagnoses can lead to more effective, generalised interventions and support systems, improving the overall well-being and daily life of children with short stature. This comprehensive perspective ensures that essential needs are addressed universally, fostering better outcomes for this population.

In recent years, there has been a growing recognition within the paediatric healthcare community of the profound significance of HRQoL assessments. This acknowledgement has catalysed the development and validation of HRQoL measures explicitly tailored to the unique needs and experiences of children and adolescents with short-stature [1, 13, 14]. Notable examples of such specialised instruments are the QoLISSY and the PedsQL questionnaire, developed to capture the challenges and intricacies of HRQoL in this population [15, 16]. These specialised tools complement more generic measures of HRQoL, such as the widely used KIDSCREEN questionnaire, which offers a broader evaluation of the quality of life outcomes among children and adolescents [17].

However, despite the emergence of specialised instruments, research into HRQoL among paediatric patients with short stature remains a dynamic and evolving study area. One crucial aspect that necessitates continual scrutiny is the evaluation of the scales employed to capture HRQoL. The importance of evaluating these scales lies in the understanding they offer regarding the unique experiences and challenges faced by paediatric patients with short-stature [1]. Accurate measurement of HRQoL furnishes invaluable insights into the precise domains of functioning that may bear the most significant impact due to the short stature [2]. It further enables meaningful comparisons among different treatment modalities. By discerning which interventions yield the most favourable outcomes across various aspects of HRQoL, clinicians and researchers can make informed decisions regarding selecting and refining therapeutic approaches [18, 19]. Understanding the available measures, their focus and how they are adopted may be instrumental in optimising treatment strategies to facilitate the HRQoL. Furthermore, integrating HRQoL measures into clinical trials emphasises its importance in understanding how interventions affect patients’ HRQoL [18, 20]. Beyond traditional clinical endpoints, understanding the variation in HRQoL measures may offer insights into the broader impacts of treatments or interventions on patients’ quality of life.

Since the last review by Brütt et al. [1], there has been a considerable change in the treatment dynamics of short-stature paediatric patients. For example, there has been a significant shift in medication practices, with the introduction of long-acting effect medication and earlier initiation of medication usage, notably from as early as two years for ACH patients. These changes signify a critical evolution in the therapeutic landscape for paediatric patients with short stature, potentially influencing their HRQoL outcomes and how this is measured.

Reviewing existing HRQoL measures is essential as it allows a summarised understanding of how researchers adopt different scales to accommodate diagnosis and age diversity among paediatric patients with short stature. Different scales prioritise distinct aspects of HRQoL and may, therefore, be more suitable for specific demographics or diagnoses. By evaluating the features of HRQoL measures and how they are used across different age cohorts, this review will provide baseline information that allows researchers to carry out HRQoL assessments that are sensitive, inclusive, and relevant and offer insights that accurately reflect the experiences of the target population. Furthermore, the review will explore the nature of the available measures covering patient-reported and observed outcomes. Discrepancies between these outcomes may highlight areas for further investigation or intervention, guiding the development of more effective treatments and interventions. Additionally, understanding the prevalence of generic versus specific measures in research provides insights into the breadth and depth of assessments conducted, enabling researchers to tailor measures to the specific context of a study population for more meaningful and actionable results.

## Materials and methods

This systematic review aims to consolidate Patient-reported Outcome Measures (PROMs) and Observed Reported Outcome Measures (ObsROM) relevant to assessing Health-Related Quality of Life (HRQoL) in short-stature paediatric patients. The methodological framework for this review adheres to established guidelines for conducting and analysing systematic reviews, including those outlined by Booth et al. [21, 22], and Newman and Gough [23]. These guidelines provide a structured approach to the research process and offer tools to effectively synthesise evidence pertinent to decision-makers in evidence-based medicine and educational research.

In line with transparency and reporting standards, this study follows the Preferred Reporting Items for Systematic Reviews and Meta-Analyses (PRISMA) 2020 statement outlined by Page et al. [24]. The PRISMA 2020 statement includes a recommended checklist for systematic reviews, ensuring the comprehensive publication of essential information. The research approach was preregistered in the International Prospective Register of Systematic Reviews (PROSPERO) on 23. January 2024. The registration details and review protocol can be accessed on PROSPERO under CRD42024505158. However, the review protocol was not prepared.

### Inclusion and exclusion criteria

The inclusion criteria (IC) and exclusion criteria (EC) were established following the PICOS format [22], as outlined in Table 1. The study aimed to include research focusing on short-stature paediatric patients (up to 18 years old) diagnosed with Achondroplasia (ACH), Growth Hormone Deficiency (GHD), and Small-for-Gestational-Age (SGA) (IC 1). However, studies involving patient caregivers, family members, or child patients with diagnoses unrelated to short stature were excluded (EC 1).

**Table 1:** Inclusion and exclusion criteria based on the PICOS scheme

Patient	
IC 1	Short-stature paediatric patients (up to 18 years old) diagnosed with Achondroplasia (ACH), Growth Hormone Deficiency (GHD), Isolated Growth Hormone Deficiency (IGHD) or Small-for-Gestational-Age (SGA)
EC 1	patients older than 18 years, parents, siblings, or caregivers; Patients with diagnoses other than ACH, GHD, SGA (not short stature)
Intervention	
IC 2	Measurement with Validated Patient-reported outcome measures (PROMs)/ Observer-reported outcome measures (ObsROMs)
EC 2	Non validated measures
Comparator	
	Not relevant
Outcome	
IC 3	Health-related quality of life, quality of life
EC 3	oral HRQOL or None Quality of life Measures
Publication	
IC 4	Original Studies published in peer-reviewed journals with abstract, title and full text in English or German
EC 4.1	Unpublished Studies, book chapters, congress contributions, Clinical reports
EC 4.2	Full text not available
Study design	
IC 5	Quantitative studies, prospective studies, clinical studies, cross-sectional and longitudinal studies
EC 5	Qualitative Studies, Reviews, Meta-Analyses, Case-Reports

Furthermore, the selected studies needed to have examined paediatric patients using validated PROMs or ObsROMs (IC 2). Studies utilising non-validated measures were excluded (EC 2). Inclusion required that the examined study have at least one outcome related to the patient’s HRQoL or Quality of Life (IC 3). Only original studies published as peer-reviewed journal articles, with abstract, title, and full text in German or English, were considered (IC 4), and no date restrictions were applied. Unpublished studies, book chapters, congress contributions, and case reports were excluded (EC 4.1).

Additionally, studies with unavailable full texts were excluded (EC 4.2). The study design for inclusion encompassed quantitative, prospective, clinical, cross-sectional, and longitudinal studies that allowed for the evaluation of PROMs (IC 5). Conversely, studies with qualitative designs, reviews, meta-analyses, and case reports were excluded from this review (EC 5). It is worth noting that the comparison group's PICOS criterion did not apply to the current review.

### Information sources and search strategy

We conducted a comprehensive search in PubMed to identify relevant literature, encompassing publications up to 03 November 2023. Before initiating the formal search process, preliminary searches were conducted. We tested the search string in the PubMed database, refining our methodology and focus based on the results. Furthermore, we explored the PROSPERO database to avoid potential overlap with unpublished studies. The final search string, as detailed in Table 2, was employed in the PubMed databases. Additional search strategies were implemented to mitigate publication bias.

**Table 2:** Terms used in systematic database literature search

Category	Search string in English
A: Quality of Life-related terms (747,531 Results; 03 November 2023	Quality of life[MeSH Terms]) OR (Health related quality of life[MeSH Terms])) OR (Patient reported outcome measures[MeSH Terms])) OR (Mental health[MeSH Terms])) OR (Psychological well-being[MeSH Terms])) OR (Quality of life[Title/Abstract])) OR (Health related quality of life[Title/Abstract])) OR (Patient reported outcome measures[Title/Abstract])) OR (Observer report[Title/Abstract])) OR (Proxy report[Title/Abstract])) OR (Quality of life assessment[Title/Abstract])) OR (Quality of life survey[Title/Abstract])) OR (Quality of life questionnaire[Title/Abstract])) OR (Mental health[Title/Abstract])) OR (Psychological well-being[Title/Abstract])) OR (subjective health[Title/Abstract])) OR (psychosocial health[Title/Abstract])) OR (Self report[Title/Abstract])
B: Disease-related terms (69,725 Results; 03 November 2023)	(((((((((((((Achondroplasia[MeSH Terms]) OR (Bone Diseases, Endocrine[MeSH Terms])) OR (Dwarfism[MeSH Terms])) OR (Infant, Small for Gestational Age[MeSH Terms])) OR (Dwarfism, Pituitary[MeSH Terms])) OR (Achondroplasia[Title/Abstract])) OR (Skeletal dysplasia[Title/Abstract])) OR (Short stature[Title/Abstract])) OR (Dwarfism[Title/Abstract])) OR (SGA[Title/Abstract])) OR (Small for Gestational Age[Title/Abstract])) OR (GHD[Title/Abstract])) OR (IGHD[Title/Abstract])) OR (Growth hormone deficiency[Title/Abstract]
C: Age-related terms (2,505,117 Results; 03 November 2023)	(((((((child*[Title/Abstract]) OR (youth[Title/Abstract])) OR (teen*[Title/Abstract])) OR (adolescen*[Title/Abstract])) OR (infant*[Title/Abstract])) OR (juvenile[Title/Abstract])) OR (pediatric*[Title/Abstract])) OR (Pediatrics[MeSH Terms])

### Screening and study selection

Following the systematic search, identified citations were exported to the reference management tool EndNote 20. Subsequently, two independent reviewers thoroughly screened all titles and abstracts, adhering to the predefined inclusion and exclusion criteria. Excluded references were annotated with the specific reason for exclusion. Articles marked as “excluded” by both reviewers were eliminated from consideration. In cases where conflicting votes (e.g., ineligible vs. potentially or probably eligible) were present, the reviewers engaged in discussions until a consensus was reached.

The agreement rate between both reviewers was calculated by determining the percentage of the sum of all matching “included” and “excluded” references, with the total number of double-screened references representing 100%. The same two reviewers proceeded to screen the full texts of all articles deemed probably eligible, applying the same inclusion and exclusion criteria during this phase of the review process.

### Data extraction and synthesis

Data were extracted from the included publications using a standardised data extraction form. The extracted data included study characteristics (e.g., study design, sample size), patient characteristics (e.g., age, diagnosis), and measures of HRQoL. The data extraction form was pilot-tested on a sample of included publications to ensure reliability and validity. The extracted data were synthesised and presented in a narrative format. The results were summarised according to the measures used in the identified publications. The identified measures were described in detail and included, e.g., level of HRQoL (generic, chronic-generic, condition-specific), scales, number of items, reliability, age groups, and report-form (patient-report/observer-report).

### Quality assessment

The methodological quality assessment of the included studies was conducted using the Joanna Briggs Institute (JBI) Critical Appraisal Checklist for Studies Reporting Prevalence Data [25]. The Critical Appraisal Checklist is a comprehensive tool designed to assess the methodological quality of studies reporting prevalence data, such as cross-sectional studies and surveys. The checklist comprises nine critical criteria, evaluating aspects such as the appropriateness of the sample frame and sampling strategy, the detailed description of participants and settings, the reliability and validity of condition identification methods, and the accurate reporting of prevalence with consideration for study design. Additionally, the checklist addresses the provision of confidence intervals for prevalence estimates. It concludes with an overall judgment on the study’s reliability in estimating the population’s prevalence of the targeted condition. By systematically examining these components, the JBI checklist facilitates a rigorous evaluation of the validity and quality of prevalence studies, aiding reviewers in systematic reviews and evidence synthesis. As shown in Table 3, the results of this evaluation suggest that *k* = 23 of the reviewed articles were of high quality. At the same time, *k* = 20 had moderate quality and *k* = 10 had low quality.

**Table 3:** Adapted rating of the methodological quality of included studies (k = 53) based on Joanna Briggs Institute (JBI) Critical Appraisal Checklist

	Author	Clear RQ^k^	Appropriate sample frame^a^	Sampling method^b^	Sample size^c^	The subject and setting adequately described^d^	Data analysis has sufficient coverage^e^	Condition identification^f^	Condition measured^g^	Appropriate statistical analysis^h^	Response rate adequate^i^	Overall quality assessment
1	Apajasalo et al. [40]^j^	+	−	+	−	+	−	+	+	+	−	LOW
2	Attanasio et al. [8]^j^	+	−	0	−	+	−	+	+	+	+	LOW
3	Bannink et al. [31]	+	0	0	+	+	+	+	+	+	+	MEDIUM
4	Bannink et al. [14]	+	+	+	+	+	+	+	+	+	+	HIGH
5	Batıbay et al. [32]	+	0	+	+	+	+	+	0	+	+	MEDIUM
6	Bloemeke et al. [13]	+	+	+	+	+	+	+	+	+	+	HIGH
7	Bloemeke et al. [41]	+	+	0	−	+	+	+	+	+	+	MEDIUM
8	Bloemeke et al. [42]	+	+	0	−	+	+	+	+	+	+	MEDIUM
9	Bullinger et al. [15]	+	+	+	+	+	+	+	+	+	+	HIGH
10	Bullinger et al. [43]	+	+	+	+	+	+	+	+	+	+	HIGH
11	Bullinger et al. [44]	+	+	+	+	+	+	+	+	+	+	HIGH
12	Butler et al. [45]	+	+	+	+	+	+	+	+	+	+	HIGH
13	Chaplin et al. [46]	+	+	+	+	−	+	+	+	+	+	MEDIUM
14	Christensen et al. [47]	+	−	−	+	+	+	+	+	+	+	MEDIUM
15	Coutant et al. [48]	+	+	0	+	−	+	+	+	+	+	MEDIUM
16	Drosatou et al. [49]	+	+	+	+	+	+	+	+	+	+	HIGH
17	Geisler et al. [50]	+	+	+	+	+	+	+	+	+	+	HIGH
18	Gizli Çoban et al. [51]	+	+	+	+	+	+	+	+	+	+	HIGH
19	Goedegebuure et al. [52]^j^	+	−	−	+	+	0	+	−	+	+	LOW
20	González Briceño et al. [34]	+	+	+	+	−	+	+	+	+	+	MEDIUM
21	Gonzalez-Briceño et al. [53]	−	+	−	+	+	−	+	+	−	+	LOW
22	Hilczer et al. [54]^j^	+	−	+	−	−	+	−	+	+	−	LOW
23	Ho et al. [55]^j^	+	+	+	+	+	+	+	+	+	−	MEDIUM
24	Kim et al. [56]^j^	+	−	+	+	+	+	+	+	+	+	MEDIUM
25	Lem et al. [57]	+	+	+	+	+	+	+	+	+	0	MEDIUM
26	Lorne et al. [58]	+	+	+	+	+	+	+	+	+	+	HIGH
27	Maghnie et al. [59]	+	+	+	+	+	0	+	+	+	0	MEDIUM
28	Maghnie et al. [60]^j^	+	−	−	−	+	+	+	+	+	+	LOW
29	Matsushita et al. [61]^j^	+	−	+	+	+	+	+	0	+	+	MEDIUM
30	Mergulhão et al. [62]	+	+	+	+	+	+	+	+	+	+	HIGH
31	Oswiecimska et al. [63]^j^	+	−	−	−	+	+	+	+	+	+	LOW
32	Otero et al. [33]	+	+	+	+	+	+	+	+	+	+	HIGH
33	Quitmann et al. [64]	+	+	+	+	+	+	+	+	+	+	HIGH
34	Quitmann et al. [4]	+	+	+	+	+	+	+	+	+	+	HIGH
35	Quitmann et al. [65]	+	+	+	+	+	+	+	+	+	+	HIGH
36	Quitmann et al. [66]	+	+	+	+	+	+	+	+	+	+	HIGH
37	Quitmann et al. [67]	+	+	+	+	+	+	+	+	+	+	HIGH
38	Rohenkohl et al. [68]	+	+	0	+	+	+	+	+	+	0	MEDIUM
39	Rohenkohl et al. [69]	+	+	+	−	+	+	+	0	+	0	MEDIUM
40	Rohenkohl et al. [70]	+	+	+	+	+	+	+	+	+	+	MEDIUM
41	Rohenkohl et al. [71]	+	+	+	+	+	+	+	+	+	+	HIGH
42	Rohenkohl et al. [69]	+	+	+	+	+	+	+	+	+	0	MEDIUM
43	Sheppard et al. [72]	+	+	+	+	+	+	+	+	+	+	HIGH
44	Silva et al. [17]	+	+	+	+	+	+	+	+	+	+	HIGH
45	Silva et al. [5]	+	+	+	+	+	+	+	+	+	0	MEDIUM
46	Sommer et al. [29]	+	−	−	+	+	+	−	+	+	+	MEDIUM
47	Stephen et al. [73]^j^	+	−	−	−	+	+	+	+	+	+	LOW
48	Stheneur et al. [74]	+	−	+	+	−	+	+	+	+	+	MEDIUM
49	Stouthart et al. [75]^j^	+	−	−	−	+	+	+	+	+	0	LOW
50	Wamstad et al. [76]^j^	+	−	−	−	+	+	+	+	+	0	LOW
51	Witt et al. [77]	+	+	+	+	+	+	+	+	+	+	HIGH
52	Wu et al. [78]	+	+	+	+	+	+	+	+	+	+	HIGH
53	Yang et al. [79]	+	+	+	+	+	+	+	+	+	+	HIGH

+ quality criterion met, – quality criterion not met, 0 = not enough information available

Overall assessment of methodological study quality: HIGH = both screening questions and all quality criteria met, MEDIUM = both screening questions met and 1-2 quality criteria not met/not enough information available, LOW = at least one screening question not met or more than 2 quality criteria not met/not enough information available

^a^Was the sample frame appropriate for the target population?

^b^Were study participants sampled appropriately?

^c^Was the sample size adequate?

^d^Were the study subjects and the setting described in detail?

^e^Was the data analysis conducted with sufficient coverage of the identified sample?

^f^Were valid methods used to identify the condition?

^g^Was the condition measured in a standard, reliable way for all participants?

^h^Was there appropriate statistical analysis?

^i^Was the response rate adequate, and if not, was the low response rate managed appropriately?

^j^The Study includes adolescent and adult samples

^k^Are there clear research questions (RQ)?

## Results

### Study Selection

**Fig. 1:**
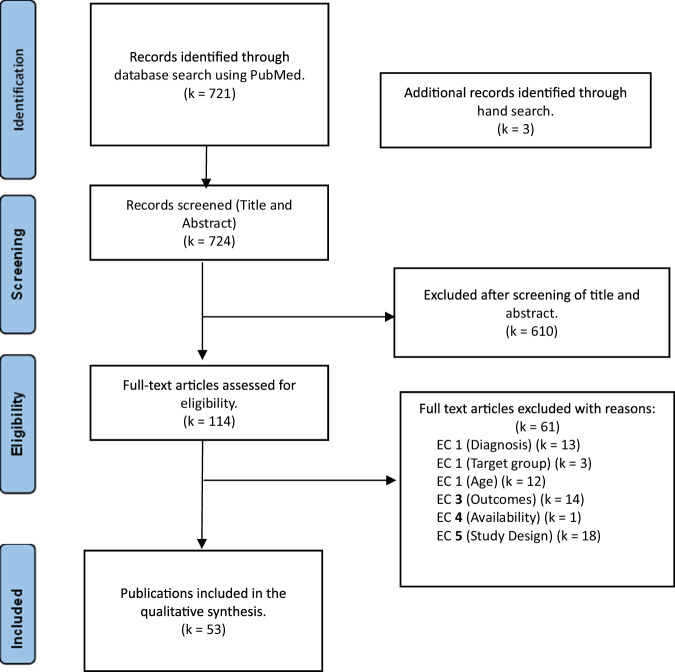
PRISMA-Flow-Diagram for the presentation of the study selection process [24]

The PubMed database was searched, identifying k = 721 publications. Additional search strategies yielded further k = 3 references. Two independent researchers screened titles and abstracts of the remaining k = 724 studies. Records not meeting the inclusion criteria were excluded (k = 610). Two independent researchers screened the remaining k = 114 full texts to assess eligibility. At the end of the study selection process, k = 54 records were included in the systematic review. The excluded records (k = 61) after full-text screening were due to study design (k = 18), diagnosis (k = 13), Target group (k = 3), age (k = 12), observed outcomes (k = 14) and unavailability of full text (k = 1). Figure 1 shows the study selection process for this systematic review in the PRISMA flow chat.

## Study characteristics

The systematic review analyses studies published between 1998 and 2023, sourced from diverse regions, including Germany (k = 7), Netherlands (k = 2), France (k = 4), United Kingdom (k = 3), China (k = 2), Turkey (k = 2), USA (k = 2), Japan (k = 1), and six additional European countries. Among the included studies, 14 were collaborative studies, notably between Germany and Spain (k = 2), Germany and Portugal (k = 3), Germany and the USA (k = 1), and among other European nations (k = 6). Most studies (k = 31) adopted a cross-sectional design, while a subset (k = 14) employed a longitudinal approach. Within the longitudinal studies, eight were observational, and seven were intervention-based. Additionally, a subgroup of 14 studies focused on validating questionnaires, while nine studies incorporated a control group into their methodologies. The list of articles reviewed is presented in Table 4 below.

**Table 4:** Reviewed publications, Title and Author and year of publication

SN	Author	Link to reference	Title of publication
1	Apajasalo et al.	[40]	Health-related quality of life of patients with genetic skeletal dysplasias.
2	Attanasio et al.	[8]	Quality of Life in Childhood-Onset Growth HormoneDeficient Patients in the Transition Phase from Childhood to Adulthood.
3	Bannink et al.	[31]	Adult height and health-related quality of life after growth hormone therapy in small for gestational age subjects.
4	Bannink et al.	[14]	Quality of Life in Adolescents Born Small for Gestational Age: Does Growth Hormone Make a Difference?
5	Batıbay et al.	[32]	Quality of Life Evaluation Following Limb Lengthening Surgery in Patients with Achondroplasia.
6	Bloemeke et al.	[13]	Psychometric properties of the quality of life in short-statured youth (QoLISSY) questionnaire within the course of growth hormone treatment.
7	Bloemeke et al.	[41]	Piloting and psychometric properties of a patient-reported outcome instrument for young people with achondroplasia based on the International Classification of Functioning Disability and Health
8	Bloemeke et al.	[42]	Cross-cultural selection and validation of instruments to assess Patient-reported outcomes in children and adolescents with achondroplasia.
9	Bullinger et al.	[15]	Cross-Cultural Equivalence of the Patient- and Parent-Reported Quality of Life in Short Stature Youth (QoLISSY) Questionnaire.
10	Bullinger et al.	[43]	Assessing the quality of life of health-referred children and adolescents with short stature: development and psychometric testing of the QoLISSY instrument.
11	Bullinger et al.	[44]	Evaluation of the American-English Quality of Life in Short Stature Youth (QoLISSY) questionnaire in the United States.
12	Butler et al.	[45]	Growth hormone treatment and health‐related quality of life in children and adolescents: A national, prospective, one‐year controlled study.
13	Chaplin et al.	[46]	Improvements in behaviour and self-esteem following growth hormone treatment in short prepubertal children.
14	Christensen et al.	[47]	Cost-effectiveness of somatropin for the treatment of short children born small for gestational age.
15	Coutant et al.	[48]	Treatment burden, adherence, and quality of life in children with daily GH treatment in France.
16	Drosatou et al.	[49]	Validation of the Greek version of the Quality of Life in Short Stature Youth (QoLISSY) questionnaire
17	Geisler et al.	[50]	Quality of Life in Children and Adolescents with Growth Hormone Deficiency
18	Gizli Çoban et al.	[51]	Psychiatric Disorders and Peer-Victimization in Children and Adolescents With Growth Hormone Deficiency.
19	Goedegebuure et al.	[52]	Cognition, Health-Related Quality of Life, and Psychosocial Functioning After GH/GnRHa Treatment in Young Adults Born SGA.
20	González Briceño et al.	[34]	Improved General and Height-Specific Quality of Life in Children With Short Stature After 1 Year on Growth Hormone.
21	Gonzalez-Briceño et al.	[53]	Adherence and quality of life in children receiving rhGH treatment.
22	Hilczer et al.	[54]	Effects of one-year low-dose growth hormone (GH) therapy on body composition, lipid profile and carbohydrate metabolism in young adults with childhood-onset severe GH deficiency confirmed after completion of growth promotion.
23	Ho et al.	[55]	Living With Achondroplasia: Quality of Life Evaluation Following Cervico-Medullary Decompression
24	Kim et al.	[56]	Is Bilateral Lower Limb Lengthening Appropriate for Achondroplasia?
25	Lem et al.	[57]	Health-Related Quality of Life in Short Children Born Small for Gestational Age: Effects of Growth Hormone Treatment and Postponement of Puberty.
26	Lorne et al.	[58]	Is height important for quality of life in children with skeletal dysplasias?
27	Maghnie et al.	[59]	Quality of life in children and adolescents with growth hormone deficiency and their caregivers: an Italian survey.
28	Maghnie et al.	[60]	Lifetime impact of achondroplasia study in Europe (LIAISE): findings from a multinational observational study.
29	Matsushita et al.	[61]	Physical, Mental, and Social Problems of Adolescent and Adult Patients with Achondroplasia.
30	Mergulhão et al.	[62]	Quality of Life of Children and Adolescents with Short Stature: The Twofold Contribution of Physical Growth and Adaptive Height-Related Cognitive Beliefs.
31	Oswiecimska et al.	[63]	Quality of life in transition phase in adolescents and young adults with severe and partial growth hormone deficiency.
32	Otero et al.	[33]	Implications of parent and child quality of life assessments for decisions about growth hormone treatment in eligible children.
33	Quitmann et al.	[64]	Parental perception of health-related quality of life in children and adolescents with short stature: literature review and introduction of the parent-reported QoLISSY instrument.
34	Quitmann et al.	[4]	Associations between Psychological Problems and Quality of Life in Paediatric Short Stature from Patients’ and Parents’ Perspectives.
35	Quitmann et al.	[65]	Validation of the Italian Quality of Life in Short Stature Youth (QoLISSY) questionnaire
36	Quitmann et al.	[66]	First-year predictors of health-related quality of life changes in short-statured children treated with human growth hormone.
37	Quitmann et al.	[67]	Quality of Life of Short-Statured Children Born Small for Gestational Age or Idiopathic Growth Hormone Deficiency Within 1 Year of Growth Hormone Treatment.
38	Rohenkohl et al.	[68]	Validation of the Flemish version of the Quality of Life in Short Stature Youth (QoLISSY) questionnaire
39	Rohenkohl et al.	[69]	Psychometric performance of the Quality of Life in Short Stature Youth (QoLISSY) questionnaire in the Netherlands.
40	Rohenkohl et al.	[70]	[Quality of life in children, adolescents, and young adults with achondroplasia]. Lebensqualität bei Kindern, Jugendlichen und jungen Erwachsenen mit Achondroplasie.
41	Rohenkohl et al.	[71]	[Living with achondroplasia- how do young persons with disproportional short stature rate their quality of life, and which factors are associated with quality of life?]. Leben mit Achondroplasie - Wie beurteilen junge Menschen mit disproportioniertem Kleinwuchs ihre Lebensqualitätund mit welchen Faktoren ist sie assoziiert?
42	Rohenkohl et al.	[80]	[Evaluation of a Self-Help Supported Counseling Concept for Children and Adolescents with Disproportional Short Stature]. Evaluation eines selbsthilfegestützten psychosozialen Interventionsangebotes für Kinder und Jugendliche mit disproportionalem Kleinwuchs.
43	Sheppard et al.	[72]	The effects of growth hormone treatment on health-related quality of life in children.
44	Silva et al.	[17]	Health-Related Quality of Life of European Children and Adolescents with Short Stature as Assessed with Generic (KIDSCREEN) and Chronic-Generic (DISABKIDS) Instruments.
45	Silva et al.	[5]	Children's psychosocial functioning and parents' quality of life in paediatric short stature: The mediating role of caregiving stress.
46	Sommer et al.	[29]	The psychometric evaluation of the quality of life in short-stature youth (QoLISSY) instrument for German children born small for gestational age.
47	Stephen et al.	[73]	Health-related quality of life and cognitive functioning in paediatric short stature: comparison of growth-hormone-naïve, growth-hormone-treated, and healthy samples.
48	Stheneur et al.	[74]	Experience of Adolescence in Patients Treated With GH during Childhood.
49	Stouthart et al.	[75]	Quality of life of growth hormone (GH) deficient young adults during discontinuation and restart of GH therapy.
50	Wamstad et al.	[76]	Neuropsychological recovery and quality-of-life in children and adolescents with growth hormone deficiency following TBI: a preliminary study.
51	Witt et al.	[77]	Quality of life of children with achondroplasia and their parents - a German cross-sectional study.
52	Wu et al.	[78]	Psychometric properties of the Chinese version of the paediatric quality of life inventory 4.0 Generic core scales among children with short stature.
53	Yang et al.	[79]	[Quality of life and its influencing factors in small for gestational age infants during early childhood].

### Samples description

The sample sizes across the included studies varied, ranging from n = 8 to n = 345 and a study with n = 5510 participants. The culminating total sample is N = 11,443 participants. Approximately half of the reported samples were female, although gender information was missing in nine studies. Specifically, the included samples comprised children and adolescents diagnosed with Growth Hormone Deficiency (GHD) (k = 35), Achondroplasia (ACH) (k = 14), and Small for Gestational Age (SGA) (k = 14). Most of the studies (k = 36) focused on a single diagnosis, with the predominant emphasis on Growth Hormone Deficiency (GHD) (k = 18) and Achondroplasia (ACH) (k = 13) (see Table 5).

**Table 5:** Description and characteristics of reviewed studies (n = 53)

SN	Author	Study design	Description; Sample size (n); Age = M(SD): Range	Sample diagnosis	Included scales	Outcome scale	Report-form (PRO/ObsROMs)
1	Apajasalo et al. [40]*	Validation study	Patients with ACH, CHH, DTD; Sample size (N = 140); Age: Range = 12 - 54 years ACH; n = 14 Age: range 12–15 (n = 6) Age range 16–24 (n = 1) *Age range 25–*34 (n* = *3)* *Age range 35–*44 (n* = *2)* *Age range 45–*54 (n* = *2)*	ACH	15D; 16D	16D	PROM/ ObsROM
2	Attanasio et al. [8]*	Prospective, Longitudinal Study	Sample size (n = 66); Age: Range = 14–24 years	childhood-onset GHD	QLS-H	QLS-H	PROM
3	Bannink et al. [31]	Multicentre, double-blind, two-arm-trial comparing the effects of two dose regimens	Sample size (n = 53); Mean age at baseline = 8.1 (SD = 1.9)	SGA	CHQ-CF87; TACQOL-S-CF	CHQ-CF87; TACQOL-S-CF	PROM; PROM
4	Bannink et al. [14]	Retrospektive Cross-sectional	Sample size (n = 39) Mean age = 14.2 (SD = 1.2)	SGA	EQ-5D-5L	EQ-5D-5L	PROM/ObsROM
5	Batıbay et al. [32]	Retrospective multicenter study	Sample size (n = 49); Mean age = 14.7 (SD = 2.44) Range = 11–18 years	ACH	PedsQL	PedsQL	PROM
6	Bloemeke et al. [13]	Validation study	Sample size (N = 118); 52 parents, 66 children; IGHD at T0; Mean age = 8.09 (SD = 3.34); SGA at T0; Mean age = 6.55 (SD = 2.64); Range = 4–17 years	IGHD, SGA	KIDSCREEN-10-index; QoLISSY; QoLISSY (proxy version)	QoLISSY; QoLISSY (proxy version)	PROM; ObsROM
7	Bloemeke et al. [41]	Validation study	Sample size (N = 133); Age 5–7; n = 45; Mean age = 6.32 (SD = 0.94); Age 8–11; n = 48; Mean age = 10.08 (SD = 1.16) Age 12–14; n = 40; Mean age = 13.42 (SD = 0.93)	ACH	APLES; APLES (proxy version)	APLES; APLES (proxy version)	PROM; ObsROM
8	Bloemeke et al. [42]	Validation study	Sample size (N = 133); Age = 5–7; n = 45; Mean age = 6.32 (SD = 0.94) Age = 8–12; n = 64; Mean age = 10.68 (SD = 1.4) Age = 13-14; n = 24; Mean age = 14.05 (SD = 0.64)	ACH	KIDSCREEN-27; KIDSCREEN-27 (proxy version); PedsQL;PedsQL (proxy version); EQ-5D-Y; EQ-5D (proxy version); PedsQL (proxy version); APLES (preliminary 15-item version); QoLISSY; QoLISSY (proxy version)	QoLISSY; QoLISSY (proxy version); APLES (preliminary 15-item version)	PROM; ObsROM
9	Bullinger et al. [15]	Validation study	Sample size (N = 268) for GHD; n = 111; Age: Range = 8–18	GHD	QoLISSY; QoLISSY (proxy version)	QoLISSY; QoLISSY (proxy version)	PROM; ObsROM
10	Bullinger et al. [43]	Validation study	Sample size N = 109; Age 8–12 (n = 40); Age 13–18 (n = 69)	GHD	KIDSCREEN-52; QoLISSY	QoLISSY	PROM
11	Bullinger et al. [44]	Validation and Evaluation Study	Sample size (N = 111) for GHD; n = 53 (25 patients, 28 parents); Age: Range = 4–18 - group 4–7 (3 parents) - group 8–12 (9 patients, 9 parents) - group 13–18 (16 patients, 16 parents)	GHD	KIDSCREEN-index; QoLISSY; QoLISSY (proxy version)	QoLISSY; QoLISSY (proxy version)	PROM; ObsROM
12	Butler et al. [45]	Prospective, one-year controlled study	Sample size (N = 189) Age: Range = 6–16 For IGHD: n = 73; For AGHD: n = 45	- IGHD (including idiopathic, isolated and multiple pituitary hormone Deficiency) - AGHD (acquired, primarily brain tumour and other oncology patients)	PedsQL	PedsQL	PROM
13	Chaplin et al. [46]	Randomised multi-centre clinical trial	Sample size (N = 99) For GHD; n = 32; Age males (n = 22); Mean age = 7.2 (SD = 1.971); Age females (n = 10); Mean age = 6.59 (SD = 1.795)	GHD	VASc; VASp	VASc; VASp	PROM; ObsROM
14	Christensen et al. [47]	Comparative Study	Sample N = 5510 SGA n = 19Mean Age 6.98 (SD = 2.24) GHD n = 5491 Mean Age 10 (SD = 1.80)	SGA, GHD	EQ-5D-5L	EQ-5D-5L	PROM
15	Coutant et al. [48]	Non-interventional, multicenter, cross-sectional study	Sample size (N = 275) For GHD; n = 166; For SGA; n = 87; Mean age = 11.5 (SD = 3.3)	GHD, SGA	PedsQl; QoLISSY; QoLISSY (proxy version)	QoLISSY; QoLISSY (proxy version)	PROM; ObsROM
16	Drosatou et al. [49]	Validation study	Sample size (N = 382); 184 children + 198 parents; For GHD; n = 165 childs + 176 parents; Age: Range = 4–18 Age = 4–7; n = 11 parents; Age 8–12; n = 73 children and 73 parents; Age 13–18; n = 92 children + 92 parents	GHD	KIDSCREEN-27; QoLISSY; QoLISSY (proxy version)	QoLISSY; QoLISSY (proxy	PROM; ObsROM
17	Geisler et al. [50]	Cross-sectional study	Sample size (N = 570); For GHD; n = 96; Mean age = 12.7 (SD = 2.4) Age: Range 8–18	GHD	KINDL	KINDL	PROM
18	Gizli Çoban et al. [51]	Cross-sectional study	Sample size (n = 61); Mean age = 12.21 (SD = 3.28) Age: Range 6–18	GHD	PedsQL; PedsQL (proxy version)	PedsQL; PedsQL (proxy version)	PROM/ObsOM
19	Goedegebuure et al. [52]*	Randomised dose-response Growth Hormone study	Sample size (n = 99) Mean age = 16+	SGA	TACQOL	TACQOL	PROM
20	González Briceño et al. [34]	Prospective, single-centre, observational cohort study	Sample size (N = 74) Mean age = 10.2 (SD = 3) Age: Range = 4.1–16.6 For GHD; n = 26 For SGA; n = 24	GHD, SGA	PedsQL; PedsQL (proxy version); QoLISSY; QoLISSY (proxy version)	PedsQL; PedsQL (proxy version); QoLISSY; QoLISSY (proxy version)	PROM; ObsROM; PROM; OBsROM
21	Gonzalez-Briceño et al. [53]	Prospective monocentric observational study	Sample size (N = 74) Mean age = 10.9 (SD = 4.1) Age: Range = 4.1–16.6 For GHD; n = 26 For SGA; n = 24	GHD, SGA	PedsQL; PedsQL (proxy version); QoLISSY; QoLISSY (proxy version)	PedsQL; PedsQL (proxy version); QoLISSY; QoLISSY (proxy version)	PROM; ObsROM; PROM; OBsROM
22	Hilczer et al. [54]*	Followup Study	Sample size (n = 54) Mean age = 17.6 (SD = 1.5)	GHD	QoL-AGHDA	QoL-AGHDA	PROM
23	Ho et al. [55]*	Monocenter cross-sectional study	Sample size (N = 109) For CMD group; n = 55 Mean age = 17.8 (SD = 11.8) For non-CDM group; n = 54; Mean age = 31.0 (SD = 16.8)	ACH	SF-36	SF-36	PROM
24	Kim et al. [56]*	Level 4 therapeutic study	Sample size (N = 44) For the treatment group, n = 22, Mean age = 12.7	ACH	SF-36	SF-36	PROM
25	Lem et al. [57]	Longitudinal intervention study	Sample size (n = 97) Mean age = 11.6 Prepubertal GH; n = 35; Mean age = 9.8 (SD = 1.1) Pubertal GH; n = 14; Mean age = 13.5 (SD = 1.1) Pub-GH+GnRHa; n = 48; Mean age = 12.3 (SD = 1.1)	SGA	TACQOL PF; TACQOL-S CF; TACQOL-S PF	TACQOL PF; TACQOL-S CF; TACQOL-S PF	ObsROM; PROM; ObsROM
26	Lorne et al. [58]	Cross-sectional study	Sample size (N = 8 families) For ACH; n = 4; Age: Range = 8–18	ACH	QoLISSY; QoLISSY (proxy version)	QoLISSY; QoLISSY (proxy version)	PROM; ObsROM
27	Maghnie et al. [59]	Cross-sectional study	Sample size (n = 142) Mean age = 12.2 (SD = 2.9) Age: Range 4–18 - for ages 4–6; n = 8 - for age 7–9; n = 16 - for age 10–12; n = 48 - for age 13–15; n = 53 - for age 16–18; n = 17	GHD	EQ-5D-3L; QoLISSY; QoLISSY (proxy version)	EQ-5D-3L; QoLISSY; QoLISSY (proxy version)	PROM/ObsROM; PROM; ObsROM
28	Maghnie et al. [60]*	Retrospective, observational study	Sample size (n = 186) Mean age = 21.7 (SD = 17.3) Age: Range 5–84.4 - for age 5–10; n = 66 - for age 11–15; n = 36 - for age 16–20; n = 17	ACH	EQ-5D-5L (proxy version); QoLISSY; QoLISSY (proxy version); PedsQL; PedsQL (proxy version)	EQ-5D-5L (proxy version); QoLISSY; QoLISSY (proxy version); PedsQL; PedsQL (proxy version)	ObsROM; PROM; ObsROM; PROM; ObsROM
29	Matsushita et al. [61]*	Cross-sectional study	Sample size (N = 130) - for ages 12–15; n = 6 - for age 16–24; n = 1	ACH	SF-36	SF-36	PROM
30	Mergulhão et al. [62]	Cross-sectional, Observational study	Sample size (n = 114) Age: Range = 8–18 For GHD; n = 33, Mean age = 13.62 (SD = 2.57); -for age 8–12; n = 10, -for age 13–18; n = 23 For SGA; n = 5	GHD SGA	QoLISSY	QoLISSY	PROM
31	Oswiecimska et al. [63]*	Cross-sectional study	Sample size (N = 76) Age: Range = 16–25 For severe GHD; n = 26; Mean age = 20.93 (SD = 0.54) Age: Range = 16.7–25.1 For partial GHD; n = 22; Mean age = 19.05 (SD = 0.43) Age: Range = 17.1–24.8	GHD	QoL-AGHDA; SF-36	QoL-AGHDA; SF-36	PROM; PROM
32	Otero et al. [33]	Cross-sectional study	Sample size (N = 144) Age: Range = 10–16 and their caregivers For IGHD; n = 67; Age: Range = 10–12 For AGHD; n = 77; Age: Range = 13–16	GHD	PedsQL; PedsQL (proxy version)	PedsQL; PedsQL (proxy version)	PROM; ObsROM
33	Quitmann et al. [64]	Psychometric Analysis/Validation study	Sample size (N = 317) for GHD; n = 130; Age: Range = 4–18 -for ages 4–7; n = 21 -for ages 8–12; n = 41 -for ages 13–18; n = 68	GHD	KIDSCREEN-52; QoLISSY; QoLISSY (proxy version)	QoLISSY; QoLISSY (proxy version)	PROM; ObsROM
34	Quitmann et al. [81]	Observational study	At baseline Sample size (N = 154) Age: Range = 4–18 For IGHD; n = 65; -for age 4–5; n = 34; -for age 8–12; n = 22; -for age 13–17; n = 9 For SGA; n = 58; Age Range = 4–7 At 1-year-follow-up Sample size (N = 130) For IGHD; n = 60; -for age 4–7; n = 26; -for age 8–12; n = 22; -for age 13–17; n = 11 For SGA; n = 48; -for age 4–7; n = 28; -for age 8–12; n = 18; -for age 13–17; n = 2	IGHD, SGA	KIDSCREEN-10-index; KIDSCREEN-52; KIDSCREEN-52 (proxy version); QoLISSY; QoLISSY (proxy version)	KIDSCREEN-52; KIDSCREEN-52 (proxy version); QoLISSY; QoLISSY (proxy version)	PROM; ObsROM; PROM; ObsROM
35	Quitmann et al. [65]	Validation study	Sample size (N = 32) Age: Range = 4–18 For GHD; n = 22; -for age 4–7; n = 3; -for age 8–12 n = 9; -for age 13–18 n = 10	GHD	QoLISSY; QoLISSY (proxy version)	QoLISSY; QoLISSY (proxy version)	PROM/ObsROM
36	Quitmann et al. [66]	Longitudinal study	Sample size (N = 111) Age: Range = 5–18 For T1 For GHD; n = 48; Mean age = 8.4 (SD = 3.32) For SGA; n = 42; Mean age = 6.9 (SD = 2.78) for T2 For GHD; n = 48; Mean age = 9.51 (SD = 3.36) For SGA; n = 42; Mean age = 8.05 (SD = 2.78)	GHD SGA	KIDSCREEN-10-index; QoLISSY; QoLISSY (proxy version)	QoLISSY; QoLISSY (proxy version)	PROM; ObsROM
37	Quitmann et al. [67]	Observational longitudinal study	Sample size (N = 154) children; Age: Range = 4–18 For Baseline - GHD; n = 65 - SGA; n = 58 T1- 1-year follow-up - GHd; n = 60 - SGA; n = 48	SGA GHD	QoLISSY; QoLISSY (proxy version)	QoLISSY; QoLISSY (proxy version)	PROM; ObsROM
38	Rohenkohl et al. [68]	Validation study	Sample size (N = 53) children; Age: Range = 4–17 For GHD; n = 12; - for age 4–7; n = 5; - for age 8–12; n = 2; - for age 13–18; n = 5	GHD	KIDSCREEN-52; QoLISSY; QoLISSY (proxy version)	KIDSCREEN-52; QoLISSY	PROM; PROM
39	Rohenkohl et al. [69]	Validation study	Sample size (N = 57) 49 children/parents +8 parents; Age: Range = 4–18 Mean Age = 11.82 (SD = 3.18) For GHD; n = 11; - for age 4–7; n = 3; - for age 8–12; n = 4; - for age 13–18; n = 4	GHD	KIDSCREEN-52; KIDSCREEN-52 (proxy version) QoLISSY; QoLISSY (proxy version); DISABKIDS-10; DISABKIDS-10 (proxy version)	KIDSCREEN-52; KIDSCREEN-52 (proxy version) QoLISSY; QoLISSY (proxy version); DISABKIDS-10; DISABKIDS-10 (proxy version)	PROM; ObsROM; PROM; ObsROM; PROM; ObsROM
40	Rohenkohl et al. [70]	Cross-sectional study	Sample size (N = 89) children; Age: Range = 8–28 + 63 parents for children; Age: Range = 8–17 - for ages 8–12; n = 23 - for ages 13–17; n = 33 - for age 18–28; n = 33	ACH	KIDSCREEN-10-index; DISABKIDS-12; QoLISSY	KIDSCREEN-10-index	PROM
41	Rohenkohl et al. [71]	Cross-sectional study	Sample size (N = 89) Age: Range = 8–28 - for ages 8–12 n = 23 - for ages 13–17 n = 33 - for age 18–28 n = 33	ACH	KIDSCREEN-52; QoLISSY; QoLISSY (proxy version)	QoLISSY; QoLISSY (proxy version)	PROM; ObsROM
42	Rohenkohl et al. [69]	Longitudinal intervention study	Sample size (n = 58) Age: Range = 8–17	ACH	QoLISSY; QoLISSY (proxy version)	QoLISSY; QoLISSY (proxy version)	PROM; ObsROM
43	Sheppard et al. [72]	Longitudinal study	Sample size (n = 22) Age: Range = 8–16	GHD	PedsQL; PedsQL (proxy version)	PedsQL; PedsQL (proxy version)	PROM; ObsROM
44	Silva et al. [17]	Cross-sectional study	Sample size (N = 208) (110 children and adolescents) For GHD, n = 59 (28 children and 31 adolescents)	GHD	KIDSCREEN-10-index; KIDSCREEN-10-index (proxy version); DISABKIDS-12; DISABKIDS-12 (proxy version)	KIDSCREEN-10-index; KIDSCREEN-10-index (proxy version); DISABKIDS-12; DISABKIDS-12 (proxy version)	PROM; ObsROM; PROM; ObsROM
45	Silva et al. [5]	Cross-sectional study	Sample size (N = 238) parent/child-dyads; Age: Range = 8–18 For GHD; n = 99	GHD	QoLISSY	QoLISSY	PROM
46	Sommer et al. [29]	Validation, psychometric evaluation study	Sample size (65 families: 17 child reports, 64 parent reports) Age: Range = 4–18	SGA	KIDSCREEN-10-index; KIDSCREEN-10-index (proxy version); QoLISSY; QoLISSY (proxy version)	KIDSCREEN-10-index; KIDSCREEN-10-index (proxy version); QoLISSY; QoLISSY (proxy version)	PROM; ObsROM; PROM; ObsROM
47	Stephen et al. [73]*	Cross-sectional study	Sample size (N = 89) For GHD; n = 23	GHD	PedsQL; PedsQL (proxy version)	PedsQL; PedsQL (proxy version)	PROM; ObsROM
48	Stheneur et al. [74]	Cross-sectional study	Sample size (n = 35) Mean age = 20.5 (SD = 4.9) Age: Range = 14.8 – 32	GHD SGA	SF-36; QLS; QLS-H	QLS-H	PROM
49	Stouthart et al. [75]*	Longitudinal intervention study	Sample size (n = 22) Age: Range = 15–22 For IGHD; n = 11; Mean age = 18.5 (SD = 2.7) For MPHD; n = 11; Mean age = 18.8 (SD = 1.1)	GHD	QLS-H	QLS-H	PROM
50	Wamstad et al. [76]*	Cross-sectional study	Sample size (n = 32) Age range = 8 - 21 Mean age = 15.7 (SD = 3.7)	GHD	CHQ-28	CHQ-28	ObsROM
51	Witt et al. [77]	Cross-sectional study	Sample size (n = 47 children, 73 parents) Age: Range = 4–14	ACH	PedsQL; PedsQL (proxy version)	PedsQL; PedsQL (proxy version)	PROM; ObsROM
52	Wu et al. [78]	Validation study	Sample size (n = 201) Age: Range = 8–18 For GHD; n = 72 For SGA; n = 10	GHD SGA	PedsQL; PedsQL (proxy version)	PedsQL; PedsQL (proxy version)	PROM; ObsROM
53	Yang et al. [79]	Randomised control study	Sample size (N = 333) For SGA; n = 203 Age: Range = 1–3	SGA	ITQOL-SF47	ITQOL-SF47	ObsROM

*The Study includes adolescent and adult samples

### Measures of health-related quality of life (HRQoL)

As presented in Table 6, the reviewed article used 25 distinct HRQoL measures in the context of short-stature paediatric patients. Of these measures, *k* = 14 were observed as outcome variables. At the same time, the remaining were employed as cross-correlates for scale validation or as predictors. Within the subset of 14 questionnaires with reported outcomes, *k* = 7 were identified as generic measures targeting HRQoL (e.g., KIDSCREEN-10), and *k* = 5 were disease-specific (e.g., QoLISSY, APLES and TACQOL). K = 1 was characterised as generic measures supplemented with disorder-specific modules (PedsQL patient report and PedsQL proxy report), and *k* = 1 was categorised as chronic generic. Additionally, among the questionnaires with reported outcomes, *k* = 13 were administered as PROMs and ObsROMs; subsequently, *k* = 3 were administered as PROMs only, and k = 1 was available in ObsROMs only. Similarly, *k* = 33 uses both PROMs and ObsROMs, *k* = 18 uses only PROMs, and *k* = 2 uses only ObsROMs.

**Table 6:** Outcome scales characteristics and distribution

SN	Outcome scales name	Scale acronym		Number of studies	The age range of participants	Diagnosis included	Report-form (PROMs/ObsROMs)	Number of items and covered subscales	Covered subscales/dimensions
1	Sixteen-dimensional generic self-assessment measure for adolescents	16D	Generic	1	12–15	ACH	PROMs (ObsROMs is possible)	16	Mobility, vision, hearing, breathing, sleeping, eating, speech, excretion, school and hobbies, mental function, discomfort and symptoms, depression, distress, vitality, appearance, friends
2	Questions on Life Satisfaction-Hypopituitarism	QLS-H	Specific	1	14–19 years	GHD, SGA	PROMs	9	- Resilience - Body shape - Self-confidence - Ability to become sexually aroused - Concentration - Physical stamina - initiative/drive - ability to cope with own anger - ability to tolerate noise/disturbance
3	EuroQoL	EQ-5D-5L	Generic	3	3–20 years	GHD, SGA, ACH	PROMs/ObsROMs	Five domains, Five items per domain representing the level of severity	- Mobility - self-care - usual activities - pain/discomfort - anxiety/depression
		EQ-5D-3L	Generic	1	4–18 years	GHD	PROMs/ObsROMs	Five domains, Three items per domain representing the level of severity	- Mobility - self-care - usual activities - pain/discomfort - anxiety/depression
4	TNO-AZL Children's Quality of Life Short Stature Module	TACQOL-S	Specific	2	8–18 years	SGA	(TACQOL-S-CF) PROMs/ (TACQOL-S-PF) ObsROMs	6	Contact with adults, contact with peers, body image, physical abilities, prospects and vitality
5	Paediatric Quality of Life Inventory (PedsQL) 4.0 Generic Core Scales	PedsQL	Generic Core + Condition-specific module	11	4–20 years	SGA, ACH, GHD	PROMs/ObsROMs	23 (Core module)	Core module: Physical functioning - social functioning - emotional functioning - school functioning
6	KIDSCREEN questionnaire	KIDSCREEN-10 Global Index	Generic	3	8–18 years	SGA, ACH, GHD	PROMs/ObsROMs	10	Physical, psychological and social functioning
		KIDSCREEN-52	Generic	3	4–18 years	GHD, ACH	PROMs/ObsROMs	52	- Physical well-being - Psychological well-being -Moods and emotions - Self Perception - Autonomy - Parent relations and home life - Financial resources - Peers and social support - School environment - Bullying
7	Infant and Toddler Quality of Life Questionnaire-47	ITQOL-SF47	Generic	1	1–3 years	SGA	ObsROMs	47	- Physical abilities - Growth and development - Bodily pain - temperament and moods - Parental-impact emotional - Parental-impact time
8	Quality of Life in Short Stature Youth	QoLISSY	Specific	24	3–18 years	GHD, ACH, SGA	PROMs/ObsROMs	Core domains: 22 items; Supplementary domains: Coping (10 items), Beliefs (4 items), Treatment (14 items)	Core domains -physical - social - emotional Supplementary domains, predictors - Coping - Beliefs - Treatment
9	Achondroplasia Personal Life Experience Scale	APLES	Specific	2	5–14 years	ACH	PROMs/ObsROMs	21	- Self-perception - Friends - Recreation - School/Kindergarten - Physical
10	Well.Being Visual Analogue Scale for Short Statue Children EuroQoL	VASc/VASp	Specific	1	3–11 years	GHD	PROMs (VASc) and ObsROMs (VASp)	39	- Alertness - Self-Esteem - Mood - Elation - Stability - Vitality
11	Child Health Questionnaire	CHQ-CF87	Generic	1	10–18 years	SGA	PROMs	87	- General health perception - Physical functioning - Role Physical - Behaviour - Mental health - Role-emotional - Parent impact emotional - Family cohesion - Bodily pain - Self-esteem - Family activities - Summary scale Physical score - Summary scale Social score
12	36-Item Short Form Survey	SF-36	Generic	1	10–19 years	ACH	PROMs	36	Physical functioning, role functioning-physical, bodily pain, general health, vitality, social functioning, role functioning, emotional, and mental health
13	DISABKIDS Chronic Generic Module	DISABKIDS-10	Chronic Generic	1	8–17 years	ACH	PROMs/ObsROMs	10	- Scale: mental (Facets: independence, emotion) - Scale: social (Facets: exclusion, inclusion) - Scale: physical (Facets: limitation, medication)
		DISABKIDS-12	Chronic Generic	1	8–18 years	GHD	PROMs/ObsROMs	12	- Scale: mental (Facets: independence, emotion) - Scale: social (Facets: exclusion, inclusion) - Scale: physical (Facets: limitation, medication)
14	KINDL	KINDL	Generic	1	8–18 years	GHD	PROMs/ObsROMs	24	Physical well-being, emotional well-being, self-esteem, family, friends, and school

A total of k = 24 studies used the *Quality of Life in Short Stature Youth (QoLISSY)* questionnaire, making it the most used outcome measure of HRQoL in the reviewed studies. The QoLISSY questionnaire was developed specifically to assess HRQoL in Short Stature children and adolescents and is available in a patient-reported form for children and adolescents aged 8–18 years and an observe-report form for parents of children aged 4–18 years. The QoLISSY questionnaire’s psychometric performance has been evaluated satisfactorily in its original study (Cronbach’s α = 0.92 for total QoL score in patient-report, α = 0.95 in parent/observer-report; 2013) and later cross-cultural validation studies. Out of k = 24 studies, *k* = 4 reported exclusively patient-reported outcomes, while *k* = 20 included both patient and proxy-reported outcomes.

Similarly, k = 11 studies used the *Paediatric Quality of Life Inventory (PedsQL)* to assess HRQoL. The PedsQL was used to measure both the patients and the parent’s perceptions of the patient’s HRQoL and is composed of two parallel versions: a patient self-report form available for children aged 8–12 years and adolescents aged 13–18 years and a parent proxy-report form. The PedsQL has a generic core supplemented by a condition-specific module. The questionnaire is available for patients ages 2–18 as child self-report (Ages 5–7, 8–12, 13–18) and parent proxy-report (Ages 2–4, 5–7, 8–12, 13–18). The PedsQL has a long form with 45 items and a short form with 23 items. The PedsQL has demonstrated good psychometric performance and reliability (Cronbach’s α = 0.83 in the patient report, α = 0.86 in the parent report) (Varni et al., 1999). Out of the *k* = 11 studies, *k* = 9 reported patients reported and proxy-version outcomes, and *k* = 2 only included patient-reported outcomes.

The *KIDSCREEN* was used as an outcome measure in six studies. This generic, unidimensional HRQoL measure assesses the child (8–18 years) and parent’s perspective on a five-point Likert scale [26]. The KIDSCREEN questionnaire is available and validated in 3 formats: KIDSCREEN-52, KIDSCREEN-27 and KIDSCREEN-10-Index. The KIDSCREEN-52 allows a detailed profile of the ten dimensions of HRQoL and has Cronbach’s Alpha ranges from 0.76 und -0.89 depending on dimension. The KIDSCREEN-52 was used in *k* = 3 studies as outcome measures. From the three studies, *k* = 2 used both PROMs and ObsROMs, while one used only PROMs. The KIDSCREEN-27 is the short version of the KIDSCREEN-52 and allows for the computation of five HRQoL dimensions. This questionnaire version was not used as an outcome measure in reviewed studies. The KIDSCREEN-10-Index allows for global HRQoL measures for monitoring and screening purposes. Higher scores indicate better HRQoL. For the current review, *k* = 3 studies used the *KIDSCREEN-10*, covering physical, psychological, and social factors. Prior studies demonstrated the instrument’s good validity and reliability, with a Cronbach’s alpha of α = 0.81 for the patient report and α = 0.80 for the parent report [17]. From the three studies*, k* = *2 used both PROMs and ObsROMs*, while one used only PROMs.

Similarly, four reviewed studies used the *EuroQol (EQ-5D)* to measure HRQoL as an outcome. The EQ-5D is a patient-reported, generic HRQoL questionnaire, with a proxy-reported version for children younger than seven and a simplified patient-reported version for children aged 8–15 (EQ-5D-Y). The EQ-5D comprises five dimensions: mobility, self-care, usual activities, pain/discomfort, and anxiety/depression. Each dimension corresponds to an item that indicates respondents’ current health status on a scale with three (EQ-5D-3L) or five (EQ-5D-5L) levels, ranging from no problems to extreme problems. The EQ-5D, in its 3L and 5L format, has extensively well-established validity as an HRQOL measure across different conditions and populations (Feng et al., 2020). Out of the *k* = 4 studies, *k* = 3 reported outcomes for the patient-reported version of the EQ-5D, and *k* = 1 included outcome for the proxy version of the EQ-5D.

*The Short Form (36) Health Survey (SF-36)* is a generic, patient-reported survey of patient health that was used in k = 4 of the reviewed studies. The questionnaire is validated and available in a short format, SF-12. The 36 questions provide a profile of two health component summary measures by assessing the patient’s health status using eight dimensions.

Three of the included studies used the child form *TNO-AZL Children’s Quality of Life Short stature module (TACQOL-S-CF)*; one of the studies utilises, in addition, the TACQOL-S PF, a short stature-specific parent form (PF), and the generic TACQOL PF, a non-disease-specific PF (24 Lem). Moreover, the third study used the TACQOL in PROMs ormate. The TACQOL-S child form (CF) is a self-report questionnaire consisting of 37 items (all referring to the last few weeks) divided into five scales: physical abilities, vitality, contact with peers, contact with adults, and body image. A higher test score indicates a better QoL on that specific scale.

The *Questions on Life Satisfaction- Hypopituitarism (QLS-H)* addresses the complaints of adult patients with growth hormone deficiency. This questionnaire was used as an outcome measure in PROMs format in *k* = 3 of the reviewed studies in samples aged 14 and above. The questionnaire was validated and had a Cronbach’s alpha score of 0.87.

Also, *k* = 2 studies used the *DISABKIDS Chronic Generic Measure* outcomes. The DCGM is available in three validated versions that vary in length. The extended version, which has 37 items, focuses on the Chronic Generic Module, which describes six dimensions of HRQoL (Independence, Physical Limitation, Emotion, Social Inclusion, Social Exclusion, and Treatment). This version was not used in any of the reviewed studies. The DCGM-12 is a short form of the DCGM-37. It is an age group adapted and condition generic measurement, assessing general subjective CGQOL of children and adolescents (8 to 16 years old) with chronic conditions. This module is a questionnaire comprising 12 items (statements). This version was used in one reviewed study as both PROMs and ObsROMs. Similarly, the DCGM-10 includes ten items measuring the impact of chronic health conditions on quality of life and two items measuring the effect of treatments. One reviewed study used the DCGM-10 in both PROMs and ObsROMs format.

Another two studies used the *Achondroplasia Personal Life Experience Scale’ APLES* to measure HRQoL outcomes and functioning based on the International Classification of Functioning, Disability and Health for Children and Youth (ICF-CY). The APLES questionnaire includes dimensions on self-perception, friends, recreation, kindergarten/school, and physical function. The psychometric performance of the APLES questionnaire has been assessed as acceptable, with a Cronbach’s of α = 0.81 for patient reports and α = 0.82 for the parent reports (Bloemeke, 2017). The included studies used both patient-reported and observer-reported versions of the APLES questionnaire.

*Quality of Life Assessment of Growth Hormone Deficiency in Adults (QoL-AGHDA)* is a questionnaire designed to measure the impact of replacement GH on patients the Quality of Life Assessment of Growth Hormone Deficiency in Adults. The disease-specific questionnaire is validated and has a Cronbach’s alpha score ranging from 0.89 to 0.91. The questionnaire was used in *k* = 2 of the reviewed studies and was assessed as PROMs. The age of samples in the two studies ranged from 15 to 25 years.

Six other questionnaires were included in single studies. These questionnaires are 15D/ 16D, QLS-H, Child Health Questionnaire (CHQ), Visual Analogue Scale for Short Stature Children Parent Version (VASp) and KINDL. The *16D* is a sixteen-dimensional, generic self-assessment health-related measure (16D) developed as a variant of the *15D* for early adolescents (12–15 years; Apajasalo et al., 1996). The 16D consists of sixteen multiple-choice questions, each representing one health-related dimension: mobility, vision, hearing, breathing, sleeping, eating, speech, excretion, school and hobbies, mental function, discomfort and symptoms, depression, distress, vitality, appearance, and friends. The 16D final score represents overall HRQoL, ranging from 0 (worst possible) to 1 (best possible). The 16D has an 86–100% repeatability coefficient across all sixteen dimensions (Apajasalo et al., 1996). The *QLS-H questionnaire*, designed for patients with GHD, comprises nine items on physical skills and emotional balance. The child form *(CF87) of the Child Health Questionnaire (CHQ)* is a self-report form. It is designed to measure generic health status covering physical and psychosocial domains in children and adolescents 18. A higher test score indicates a better quality for that specific scale, with a maximum score of 100. The Infant and Toddler Quality of Life (ITQOL) complements the Child Health Questionnaire (CHQ). It is available in two versions - the 97-item full-length version (ITQOL) and the 47-item short-form (*ITQOL-SF47*) that differ in length. ITQOL-SF47 is a generic measure used as an ObsROM in one of the reviewed studies with a sample aged between 1 and 3 years. The *Visual Analogue Scale for Short Stature Children Parent Version (VASp)* has been shown to have good internal consistency and reliability, covering the following dimensions: alertness, self-esteem, mood, joy, stability, vitality and overall well-being.

Lastly, the self-reported *KINDL* questionnaire measures quality of life by including 24 items referring to six domains: physical well-being, emotional well-being, self-esteem, family, friends, and school. The scores were transformed to values between 0 and 100, with higher values indicating better HRQoL.

## Discussions

### Summary of key findings

The systematic review examined 53 records, encompassing studies published from 1998 to 2023, with a central focus on evaluating HRQoL in paediatric patients with short stature. This examination involved a rigorous selection process, which ultimately included a diverse range of research primarily conducted in European countries. The review analysed multiple HRQoL measures, illuminating the prevalent use of the QoLISSY, PedsQL, and KIDSCREEN measures. The different measures provided invaluable insights into the perception of HRQoL among paediatric patients, encompassing those diagnosed with GHD, IGHD, ACH, and SGA. The goal is to access it. Additionally, the review furnished a comprehensive overview of how these instruments were tailored for specific age cohorts and delineated the observed disparities in HRQoL outcomes. The four main findings are discussed below.

First, an examination of the instrument’s dimension included in the reviewed studies confirms the utilisation of disease-specific and generic measures in assessing HRQoL in paediatric patients with short stature. This adaptation provides valuable insights into the multifaceted nature of short-stature conditions and their impact on various aspects of quality of life. While the disease-specific measures are tailored to assess the specific symptoms, functional limitations, and psychosocial challenges associated with short stature [17, 27], these measures provide detailed information about the disease’s effects on HRQoL and are often more sensitive to changes in disease severity or treatment outcomes [14, 17]. On the other hand, generic measures of HRQoL offer a broader perspective by capturing overall HRQoL and functioning across physical, emotional, and social domains [28]. These broader conceptualisations allow for comparisons across different patient populations and facilitate the evaluation of the relative burden of short stature compared to other health conditions [29]. The widespread use of disease-specific quality-of-life measures in the reviewed studies may reflect recognising the unique challenges affected children and adolescents face and the need for targeted assessments to capture their experiences adequately [1, 30]. However, more studies’ adaptation of generic measures allows for a more comprehensive evaluation of HRQoL by capturing aspects that may not be directly related to short stature but are still significant contributors to HRQoL [31–33]. For example, the KIDSCREEN assess general physical functioning or emotional well-being, which could be affected by factors other than short stature, such as comorbidities or environmental influences [4, 26]. Therefore, the adaptation of generic measures may be justified as it provides a base for comparing the HRQoL of children and adolescents with that of the general population of another patient group.

Secondly, findings from the current review highlighted the extensive adoption of QoLISSY, PedsQL and KIDSCREEN as the most common measures of HRQoL outcomes across diverse age demographics and diagnostic classifications. The popularity of these instruments underscores their robustness and reliability in encapsulating the multifaceted dimensions of HRQoL among paediatric patients with short stature [4, 13, 34]. It also elucidated the nuanced insights these measures offer regarding the ramifications of short stature on various aspects of life, ranging from physical functionality to psychosocial aspects of HRQoL. Consequently, it imperatively acknowledges that the uniform adoption of these instruments across distinct age groups overlooks the nuanced developmental and social factors that may differentially impact HRQoL among younger children and those in different stages of childhood and adolescence (for example, school-age children) [35]. While these questionnaires may have different versions, they primarily differ in length and focus on PROMS or ObsROMs. Drawing on insights from existing literature and alternative patient cohorts, it becomes evident that individual developmental status during childhood and adolescence may exert a differential influence on how patients perceive (or do not perceive) their condition and its ramifications on their lives, subsequently affecting their subjective HRQoL [14]. Hence, adapting these measures across age categories may omit the nuanced understanding of developmental trajectories and social dynamics to comprehensively elucidate the intricate interplay between short stature and HRQoL across diverse paediatric populations.

Furthermore, the systematic review reveals intriguing trends regarding the adaptation of adult measures of HRQoL for use in adolescent populations. Notably, instruments such as SF-36, QoLAGHDA, and QLS-H, initially designed for adult populations, were frequently employed in assessing HRQoL among adolescents with short stature. While this adaptation might provide comparable results, it may also ignore the uniqueness of child and adolescent experiences that may affect their HRQoL outcomes. Moreover, the results highlight a significant gap in HRQoL assessment tools for younger paediatric populations. Astonishingly, only one questionnaire developed explicitly for children under four years was included in the review, leaving a notable void in the HRQoL assessment for this age group. Even more concerning, no questionnaire was available for infants below one-year-old, indicating a critical deficiency in addressing the HRQoL needs of the youngest paediatric patients with short stature.

The result suggests that HRQoL assessment is predominantly conducted using a combination of PROMs and ObsROMs in the studies reviewed. This approach signifies a comprehensive methodology that captures subjective patient perspectives and externally observed data, providing a more refined understanding of HRQoL [18, 36]. The integration of PROMs and ObsROMs in most of these studies suggests that researchers strive to encompass a broader spectrum of factors contributing to HRQoL. It further acknowledges the importance of both patient-reported experiences and observed outcomes in evaluating the overall health status of short-stature paediatric patients [37, 38]. However, it is worth noting that there are instances where HRQoL is assessed solely through PROMs, albeit with less frequency than the combined approach. This indicates a recognition of the ongoing significance of PROMs in HRQoL assessment while simultaneously highlighting the value attributed to including observed outcomes alongside patient-reported measures. This balanced approach underscores an appreciation of the complexity inherent in assessing HRQoL. It emphasises the necessity for methodological flexibility to capture the diverse dimensions of patients’ health experiences accurately.

Moreover, it is essential to consider demographic and health-related factors influencing the choice between PROMs and ObsROMs. Variables such as the patient’s health condition or age could impact their ability to complete questionnaires accurately, affecting the selection of assessment methods tailored to individual patient needs and circumstances. This is accounted for in some instruments; for example, the PedsQL instrument offers a self-report (PROMs) version suitable for individuals aged five years and older and a proxy report (ObsROMs) version applicable to individuals aged two years and older. This adaptability is crucial to assessing HRQoL across different developmental stages, catering to the diverse needs of short-stature paediatric patients [39].

### Methodological limitations

Despite the comprehensive approach, several methodological limitations warrant consideration. Firstly, the review relied on studies indexed in the PubMed database, potentially overlooking relevant publications from other databases or sources. This limitation may introduce selection bias, affecting the comprehensiveness of the review. Moreover, the variability in sample sizes across studies, ranging from small cohorts to larger samples, could influence the robustness and generalizability of findings. Reviewing HRQoL measures for paediatric patients with short stature without distinguishing between different diagnoses can highlight common challenges but also overlook specific medical, psychological, and social needs unique to each condition, potentially leading to inadequate measures. This approach might also dilute research findings, making addressing distinct issues within particular diagnoses harder. Including heterogeneous populations further poses challenges in synthesising results and drawing overarching conclusions. The predominance of cross-sectional designs limits the establishment of causal relationships and longitudinal insights into HRQoL dynamics over time.

### Strengths and suggestions for future research

Despite the noted limitations, the systematic review demonstrates several strengths. The rigorous screening process, involving multiple researchers’ independent assessments, enhances the included studies’ reliability and validity. The utilisation of a wide array of HRQoL measures provides a comprehensive overview of patient-reported outcomes and proxy assessments, contributing to the understanding of HRQoL in diverse populations. Several suggestions can be proposed to address the identified limitations and enhance the robustness of future research. Firstly, future reviews should adopt a more comprehensive search strategy, encompassing multiple databases and grey literature sources, to ensure the inclusivity of relevant publications.

Additionally, longitudinal studies with larger sample sizes and diverse populations could provide valuable insights into the trajectory of HRQoL outcomes and factors influencing longitudinal changes. Moreover, comparative studies examining the effectiveness of different HRQoL measures in specific populations could inform the selection of appropriate instruments for future research and clinical practice. Qualitative research approaches, such as in-depth interviews or focus group discussions, could complement quantitative assessments, providing deeper insights into the lived experiences and psychosocial determinants of HRQoL among children and adolescents with growth-related disorders. The findings underscore a critical imperative for intensified research and development directed towards age-appropriate HRQoL assessment tools tailored to the paediatric age spectrum, notably including children aged 0 to 4. Considering the varying and early onset of treatments for short stature, typically commencing from 4 months for Achondroplasia patients, it becomes even more imperative to bridge this gap in assessment tools to support the early evaluation of treatment effect on HRQoL.

## Conclusions

In conclusion, the systematic review offers valuable insights into the HRQoL perceptions among children and adolescents with growth-related disorders. Despite methodological limitations, the review underscores the importance of considering diverse HRQoL measures and populations to assess and address the HRQoL comprehensively. While disease-specific measures offer detailed insights into the unique challenges faced by paediatric patients with short stature, the incorporation of generic measures provides a broader context for understanding their overall well-being and facilitates comparisons with other patient populations. There is a need for further research to incorporate an understanding of developmental trajectories and social dynamics to capture better the complexities of HRQoL among diverse paediatric populations with short stature.

## Supplementary information

Supplementary Information
